# Supplementary material for: Selective clonal persistence of human retroviruses in vivo: Radial chromatin organization, integration site, and host transcription
Source: Sci Adv. 2022 Apr 29;8(17):eabm6210. doi: 10.1126/sciadv.abm6210 (PMC9054021; doi:10.1126/sciadv.abm6210)
Supplement: Supplementary file 1 — Figs. S1 to S14 Tables S1 to S4 [file sciadv.abm6210_sm.pdf]

Science Advances
AAAS

Supplementary Materials for

Selective clonal persistence of human retroviruses in vivo: Radial chromatin organization, integration site, and host transcription

Anat Melamed*, Tomas W. Fitzgerald, Yuchuan Wang, Jian Ma,
Ewan Birney, Charles R. M. Bangham*

*Corresponding author. Email: anat.melamed07@imperial.ac.uk (A.M.); c.bangham@imperial.ac.uk (C.R.M.B.)

Published 29 April 2022, *Sci. Adv.* **8**, eabm6210 (2022)
DOI: 10.1126/sciadv.abm6210

The PDF file includes:

Figs. S1 to S14
Tables S1 to S4
Legend for data S1

Other Supplementary Material for this manuscript includes the following:

Data S1

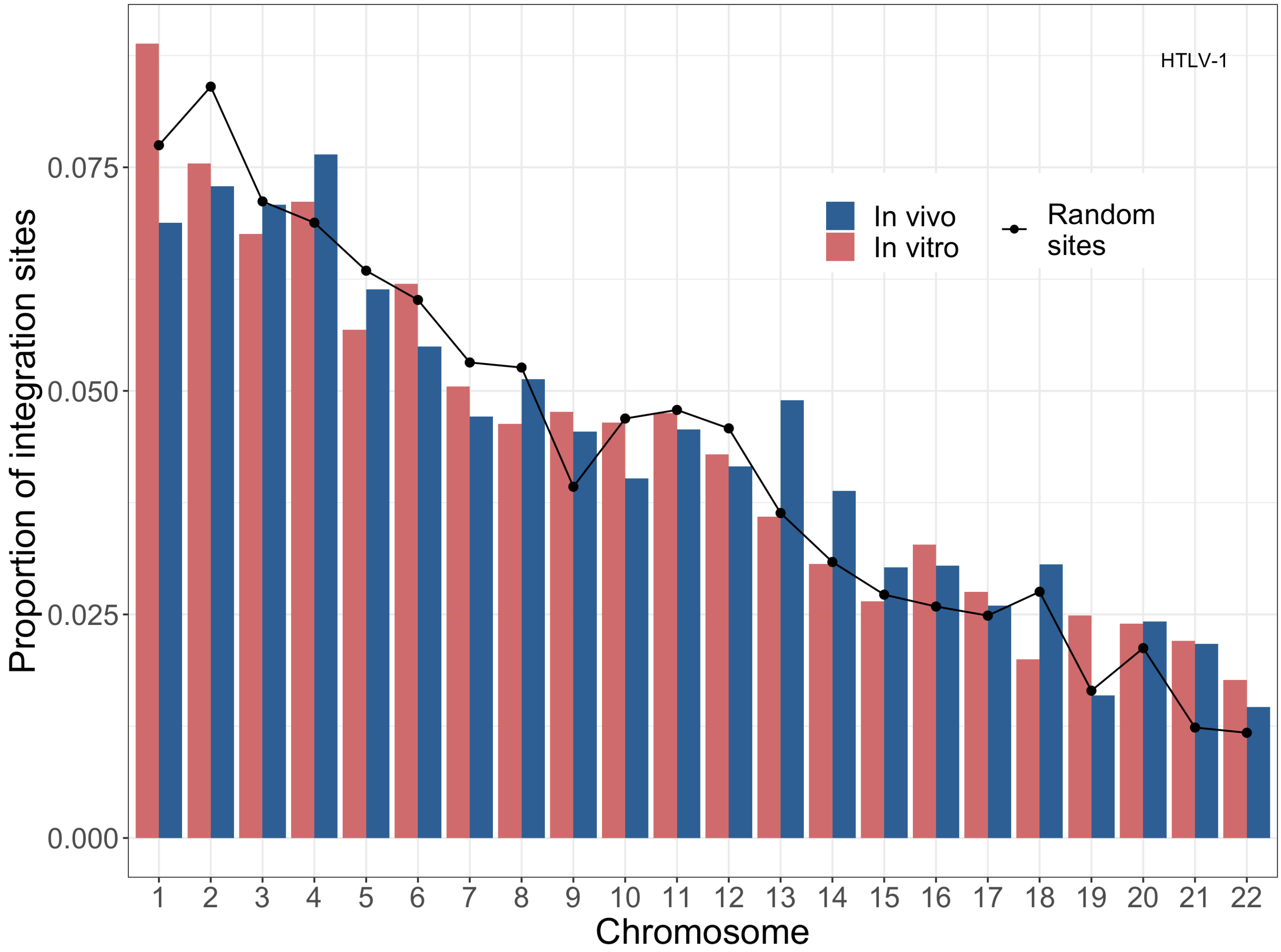

Fig. S1. HTLV-1 Integration site survival is biased to specific chromosomes. *For each chromosome, the relative frequency of integration sites present in vivo and in vitro is shown (in vivo - red, in vitro - blue). The black line shows the proportion of random sites found in each chromosome.*

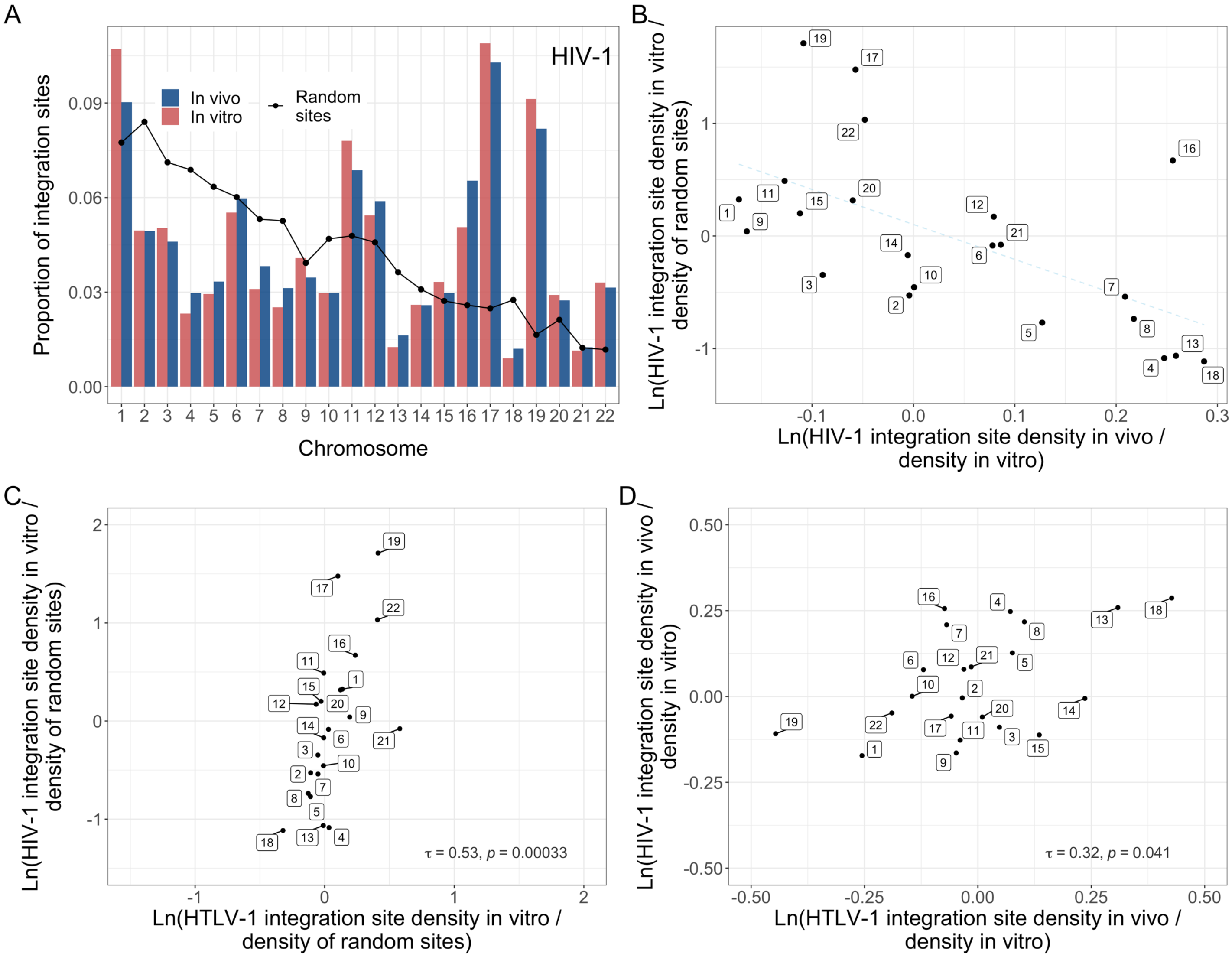

Fig. S2. HIV-1 integration site targeting and survival is biased to specific chromosomes. *For each chromosome, the relative frequency of integration sites present in the HIV-1 in vitro and HIV-1 in vivo datasets was calculated.* ***(A)*** *Integration site frequency. The black line shows the proportion of random sites found in each chromosome.* ***(B)*** *For each chromosome, two ratios of integration site frequencies (F) were calculated:* $F_{in\ vivo}$ / $F_{in\ vitro}$ *and* $F_{in\ vitro}$ / $F_{random\ sites}$ *(logarithmic scales). As in HTLV-1 (Fig. 1), HIV-1 survival is most strongly favoured in chromosome 18.* ***(C)*** *Significant positive correlation between the rank order of chromosomes favoured for initial integration between HTLV-1 and HIV-1 (Kendall's rank correlation test); the magnitude of chromosome preference in initial integration targeting is much greater in HIV-1.* ***(D)*** *Significant positive correlation between HTLV-1 and HIV-1 in the rank order of chromosomes favoured for integration site survival in vivo (Kendall's rank correlation test).*

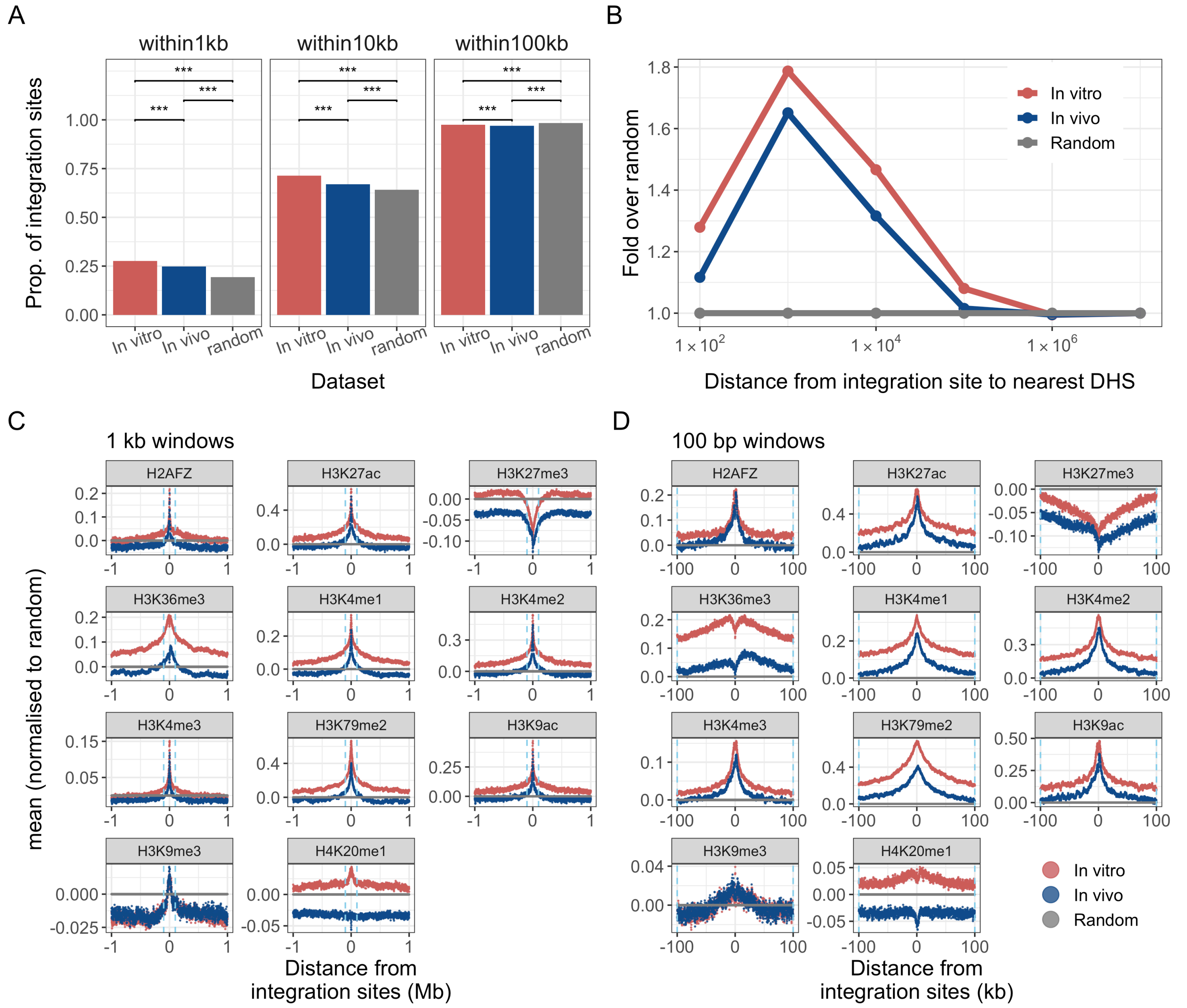

Fig. S3. Initial HTLV-1 integration is biased towards accessible, transcriptionally active chromatin. *HTLV-1 Integration site and random sites were mapped with respect to transcription factor binding sites (TFBS), DNAse hypersensitive sites (DHS) and histone marks mapped by the ENCODE project for the B cell line GM12878.* ***(A)*** *In vitro sites were annotated with respect to the distance to the nearest binding site of any of the transcription factors . HTLV-1 integration sites both in vitro and in vivo were significantly enriched within 1 kb and 10 kb of any TFBS (**** $p < 10^{-16}$; χ^2 *test with Bonferroni correction).* ***(B)*** *Integration sites were enriched within 10 kb of a DNAse hypersensitive site (DHS). In each analysis, integration site frequency is compared to a random distribution.* ***(C),(D)*** *The mean density of each of 11 histone marks flanking the integration sites was calculated in discrete windows upstream and downstream across all integration sites from each dataset, either* ***(C)*** *- 1 kb windows, up to 1 Mb from the integration site, or* ***(D)*** *- 100bp windows, up to 100 kb from the integration site.* $\text{Ln}(fold\ change\ from\ random)$ *is shown. Dashed line denotes 100 kb upstream or downstream.*

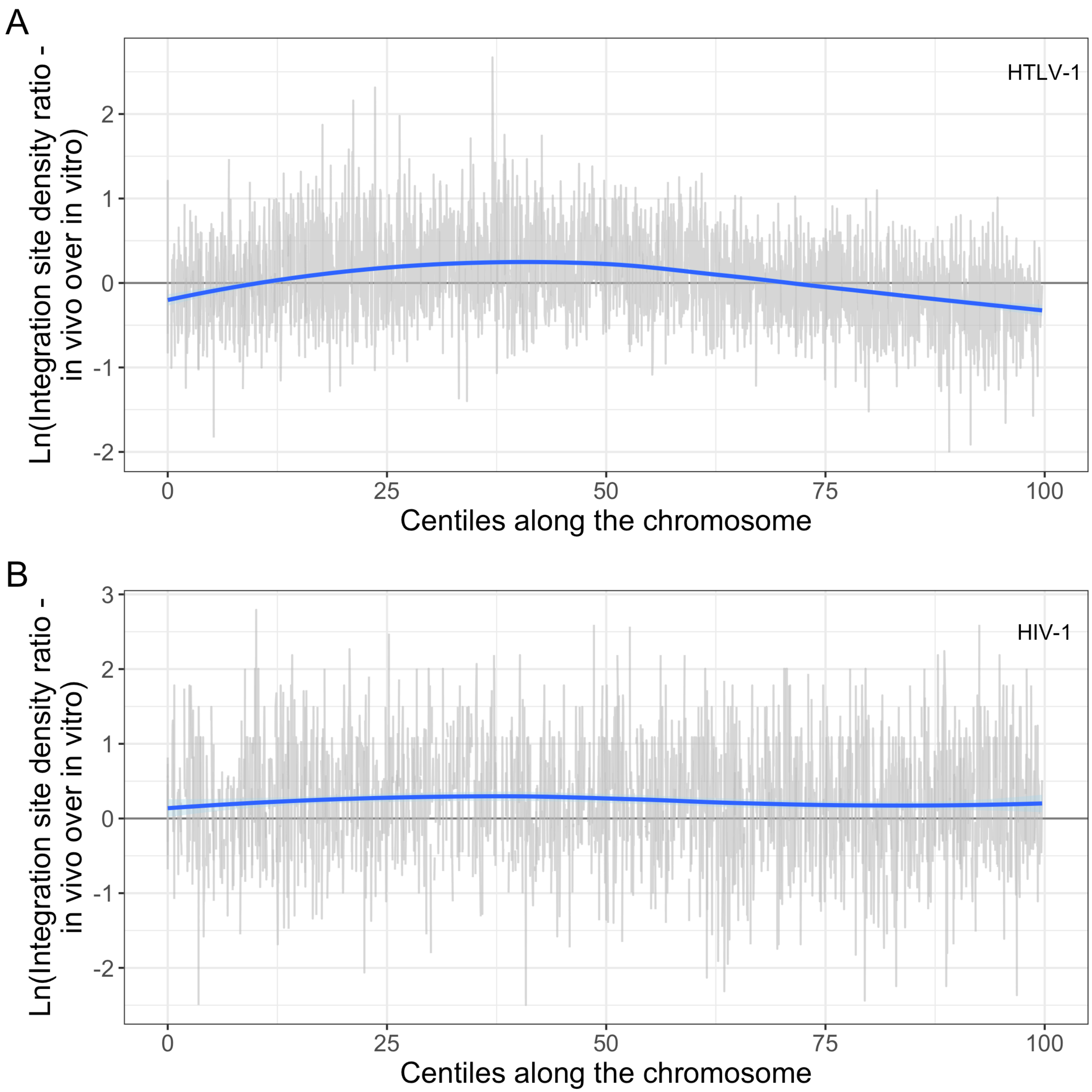

Fig. S4. HTLV-1 and HIV-1 survival vs position along the chromosome. *For each chromosome, we calculated the clone survival index (CSI; of either HTLV-1* ***(A)*** *or HIV-1* ***(B)****) in discrete 1 Mb windows. Then for each chromosome, the genomic coordinates were converted to relative centiles along the chromosome, and the CSI plotted versus this relative position. In HTLV-1 in particular there is a clear trend (trendline using LOESS method) from increased survival in the centre of the chromosome to decreased survival towards the telomeres.*

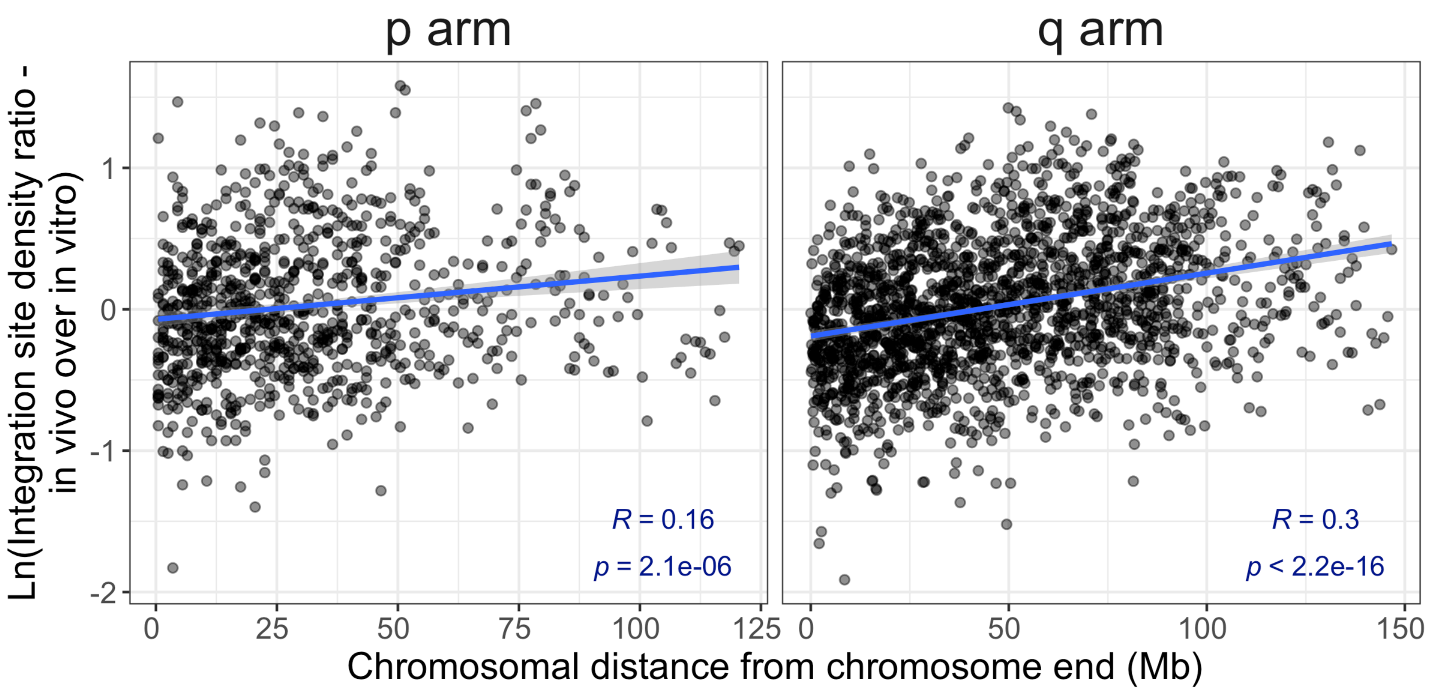

***Fig. S5. HTLV-1 survival vs distance from the telomere**. The HTLV-1 clone survival index CSI_{HTLV-1} is significantly positively correlated with the absolute genomic distance from the telomere on both short and long arms of the chromosome (Pearson's correlation test); however, this correlation is weaker than the correlation with the distance from the centromere (Fig. 3).*

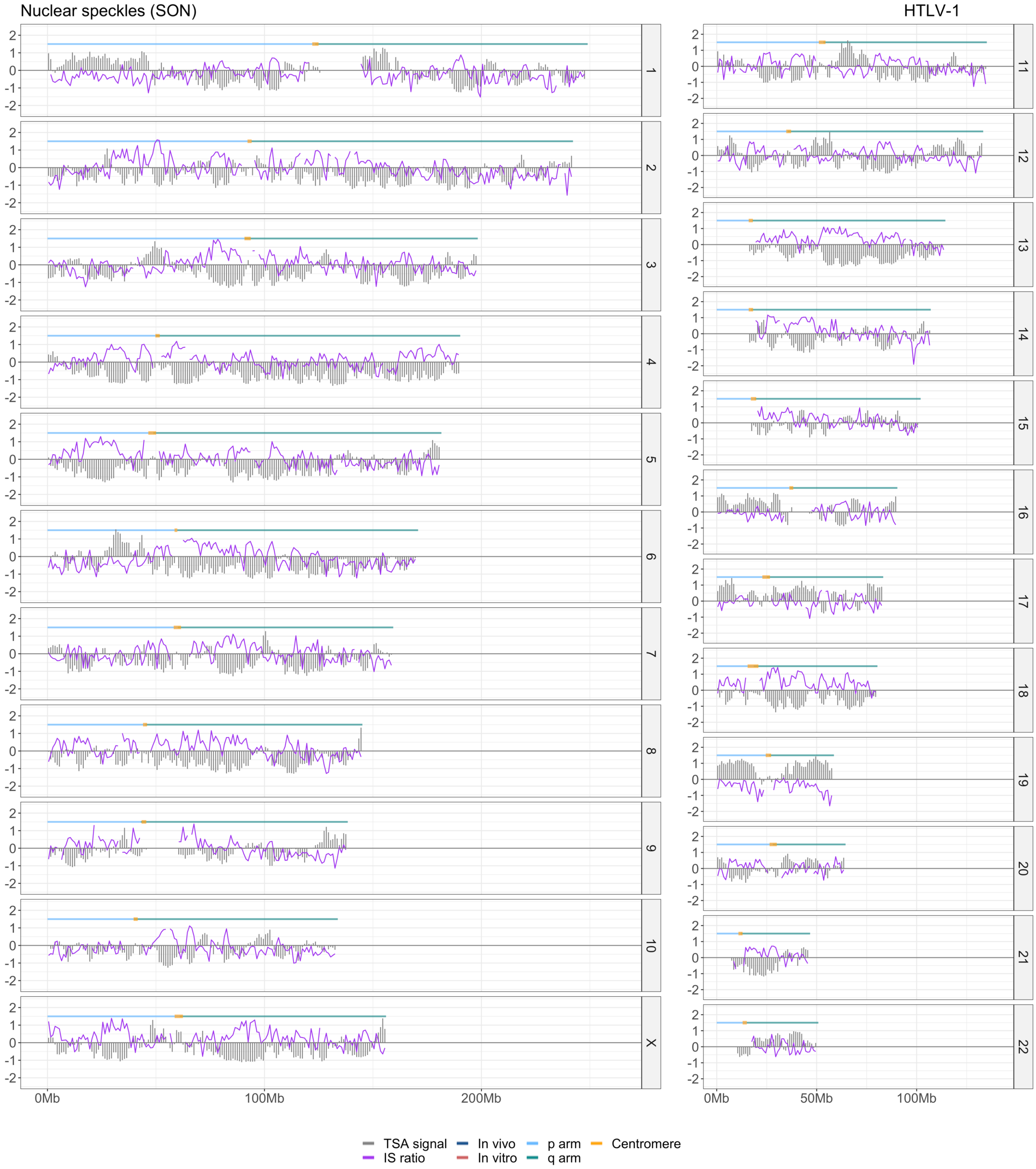

Fig. S6. HTLV-1 Clone survival index vs distance from nuclear speckles. *In each panel, the TSA-seq data for SON are plotted against* CSI_{HTLV-1} *for each chromosome. The panels for chromosomes 11, 12 are also shown in Fig. 4B, and are included here for completeness. The panel for chromosome 10 is also included in Fig. 5A.*

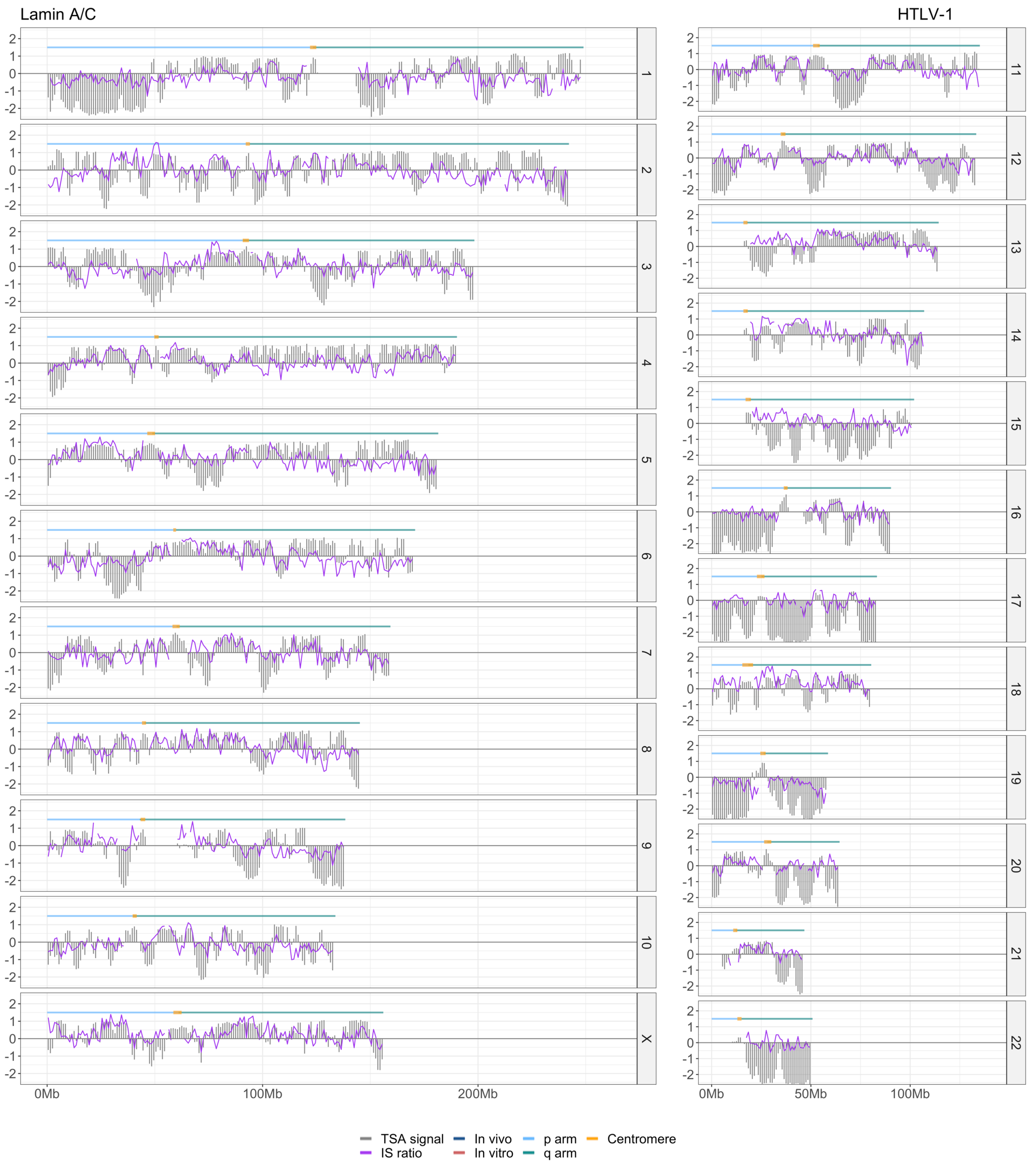

***Fig. S7. HTLV-1 Clone survival index vs distance from Lamina**. In each panel, the TSA-seq data for Lamin A/C are plotted against CSI_{HTLV-1} for each chromosome. The panels for chromosomes 11, 12 are also included in Fig. 4B. The panel for chromosome 10 is also included in Fig. 5A.*

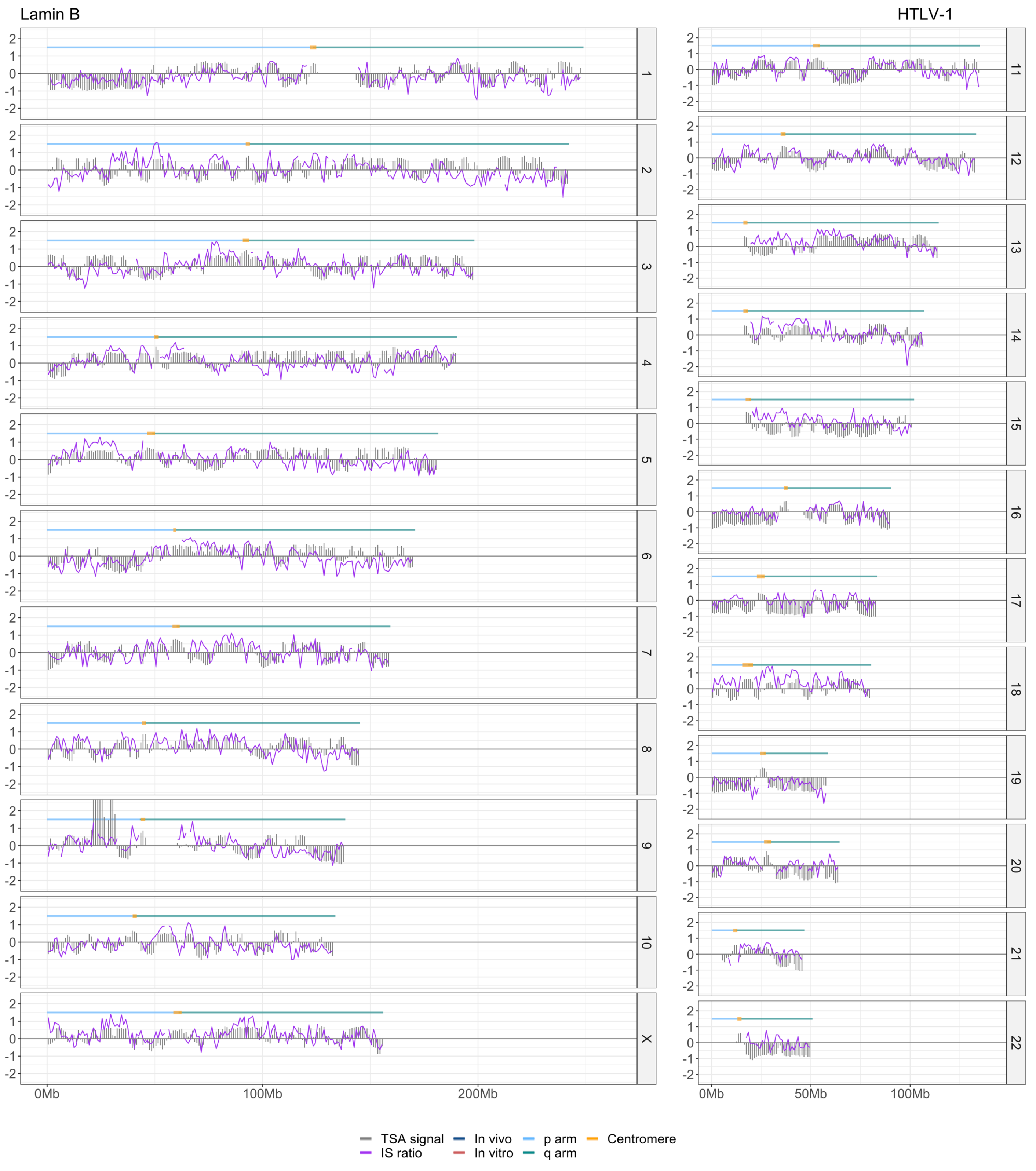

Fig. S8. HTLV-1 Clone survival index vs distance from Lamina. *In each panel, the TSA-seq data for Lamin B are plotted against CSI_{HTLV-1} for each chromosome.*

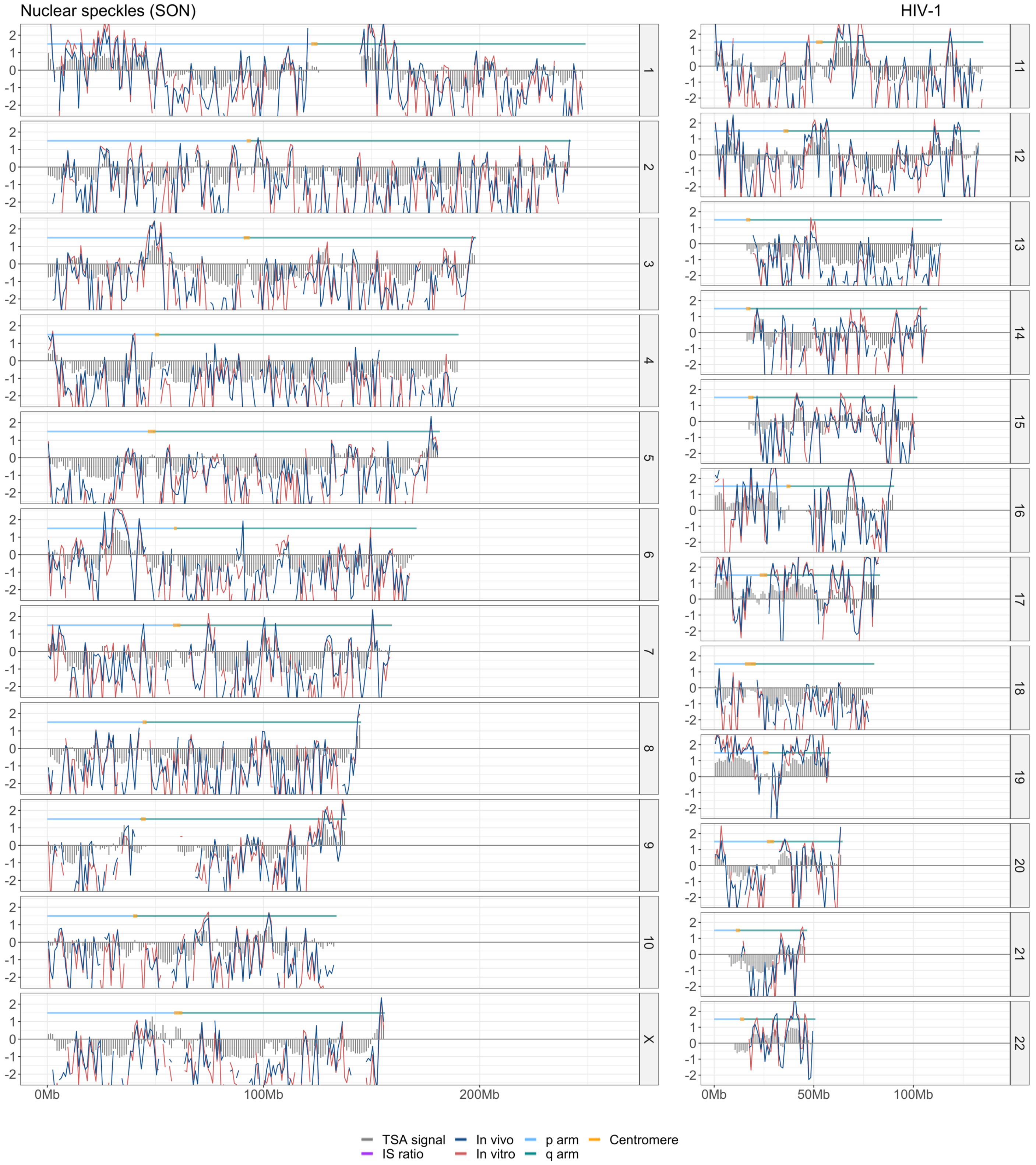

Fig. S9. HIV-1 integration site frequency vs distance from nuclear speckles. *In each panel, the TSA-seq data for SON are plotted against the integration site frequency for either HIV-1 in vitro (red) or in vivo (blue) (normalized over random) in each chromosome.*

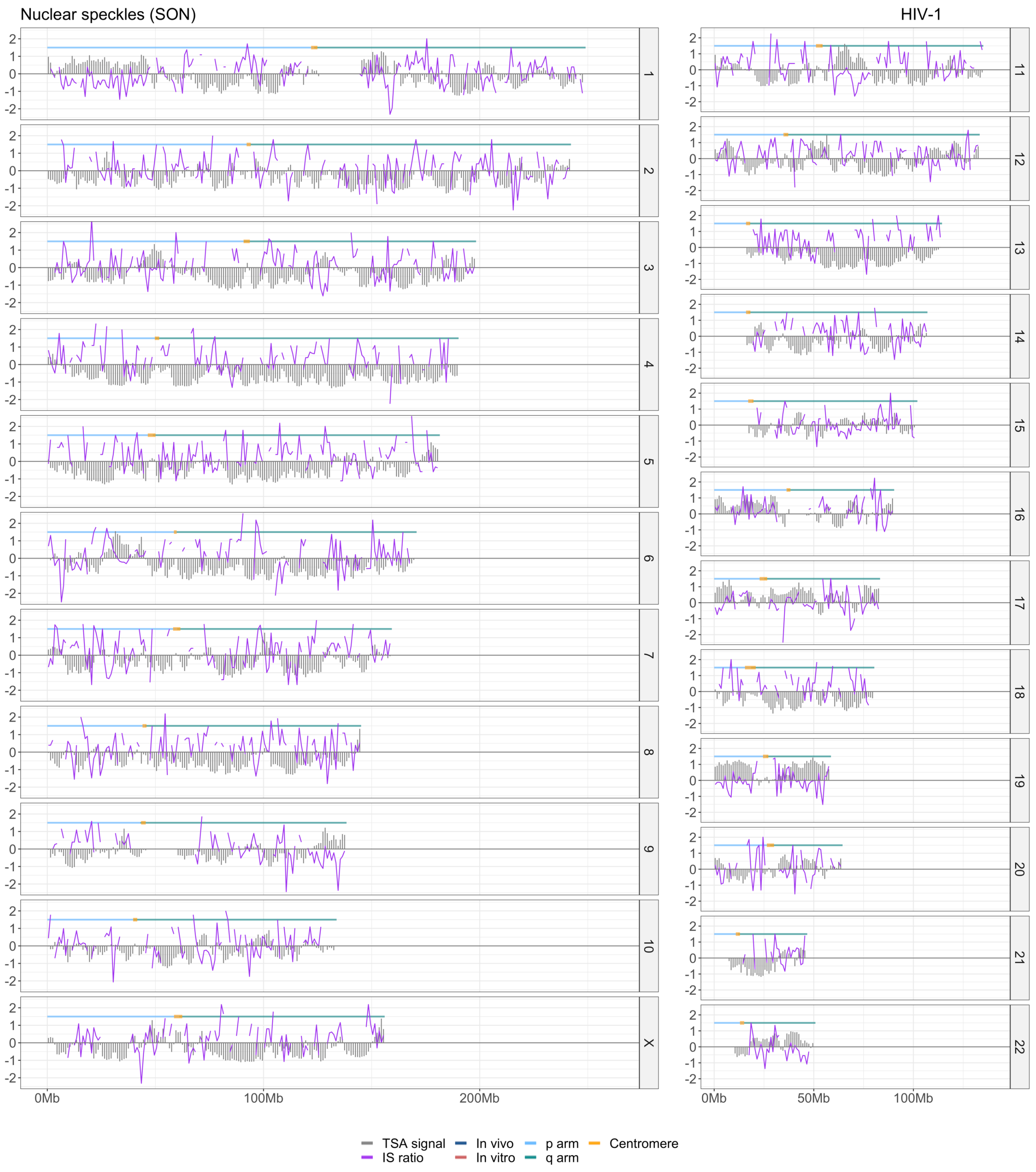

Fig. S10. HIV-1 Clone survival index vs distance from nuclear speckles. *In each panel, the TSA-seq data for SON are plotted against CSI_{HIV-1} for each chromosome. The panels for chromosomes 11, 12 are also shown in Fig. 4C, and included here for completeness.*

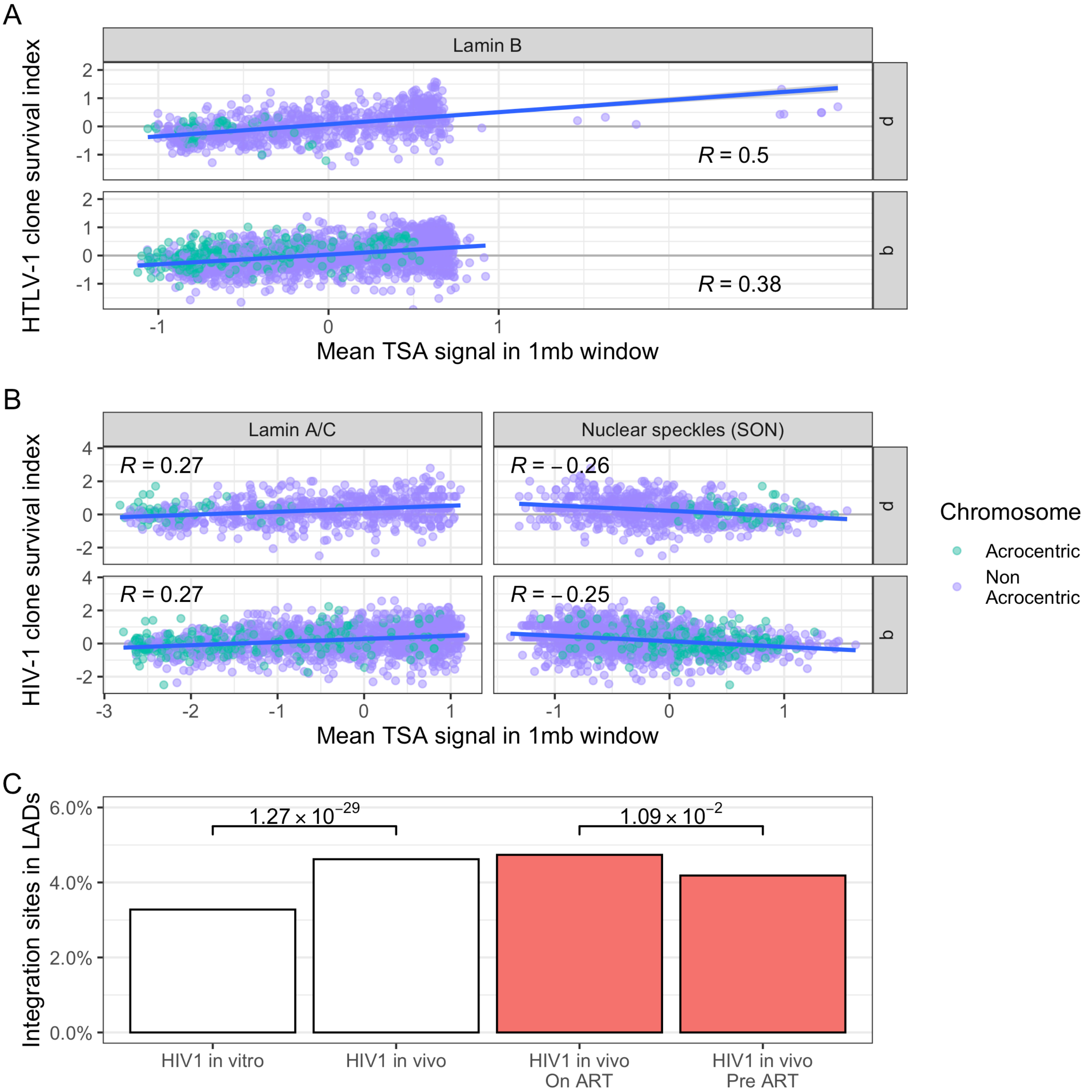

Fig. S11. Genome-wide correlation between proximity to nuclear lamina and selective clonal survival in vivo of HTLV-1 and HIV-1. (A) *TSA-seq data on Lamin-B are significantly correlated with the clone survival index across the whole genome (Pearson's correlation test,* $p < 10^{-16}$ *for each correlation).* ***(B)*** *TSA-seq data on lamin proteins and SON are significantly correlated with the clone survival index across the whole genome (Pearson's correlation test,* $p < 10^{-11}$ *for each test).* ***(C)*** *HIV-1 integration sites from individuals receiving ART are found more frequently in lamina-associated domains (LADs) than are those from individuals pre ART (Fisher's exact test). White columns (combined in vivo dataset vs in vitro data) also shown in Fig. 5 are shown here again for comparison.*

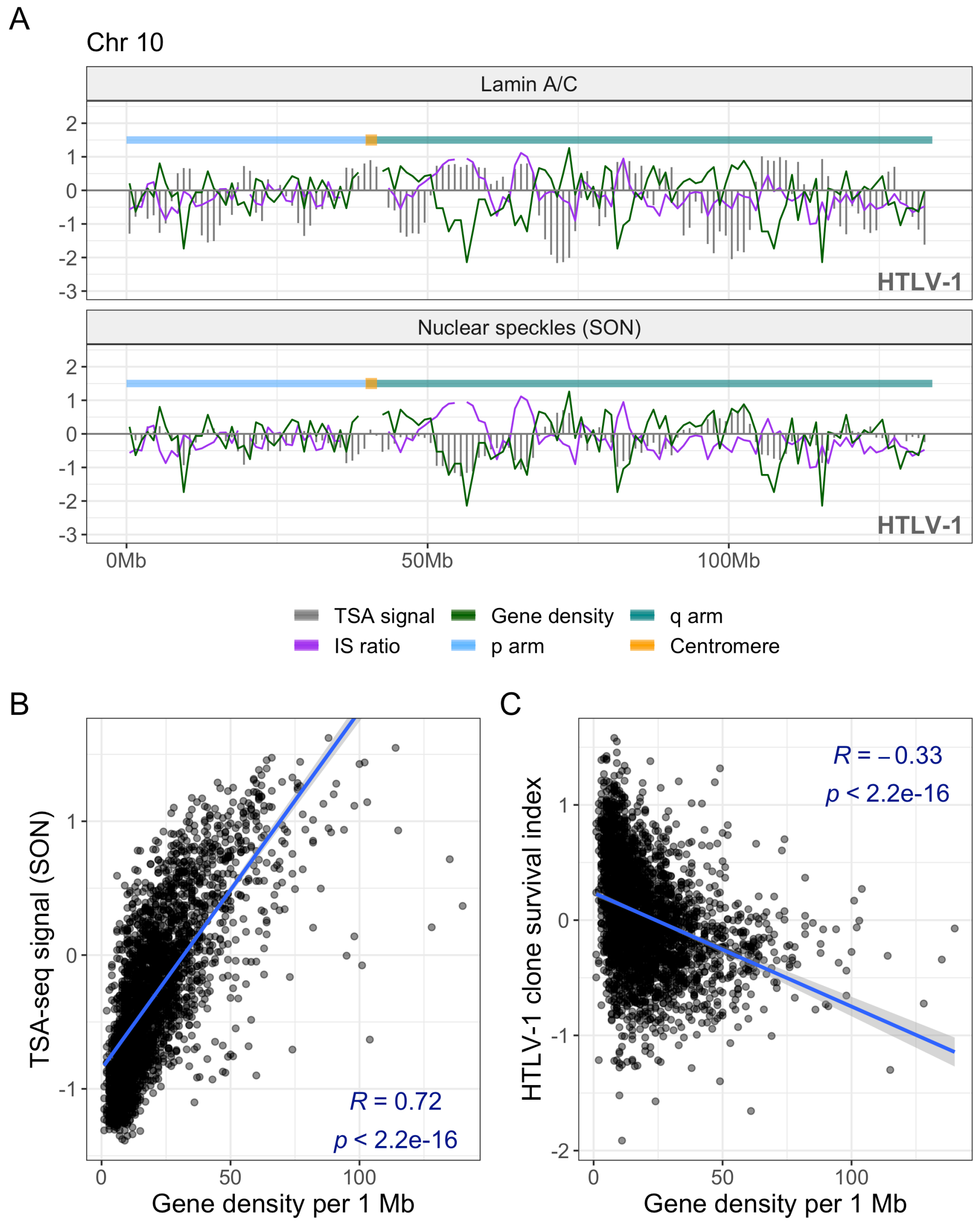

Fig. S12. HTLV-1 clone survival is reduced in gene-rich genomic regions. *The number of genes overlapping each 1 Mb window across each chromosome was quantified and compared against the HTLV-1 clone survival index.* ***(A)*** *TSA-seq data on Lamin A/C and SON from (20) are plotted against the gene density (green line) and the HTLV-1 clone survival index* CSI_{HTLV-1} *(purple). The gene density closely follows the proximity to nuclear speckles.* ***(B)*** *Genome-wide, there is a significant positive correlation (Pearson's correlation test) between the proximity to nuclear speckles and the gene density.* ***(C)*** *A significant negative correlation is observed between the HTLV-1 clone survival index and gene density.*

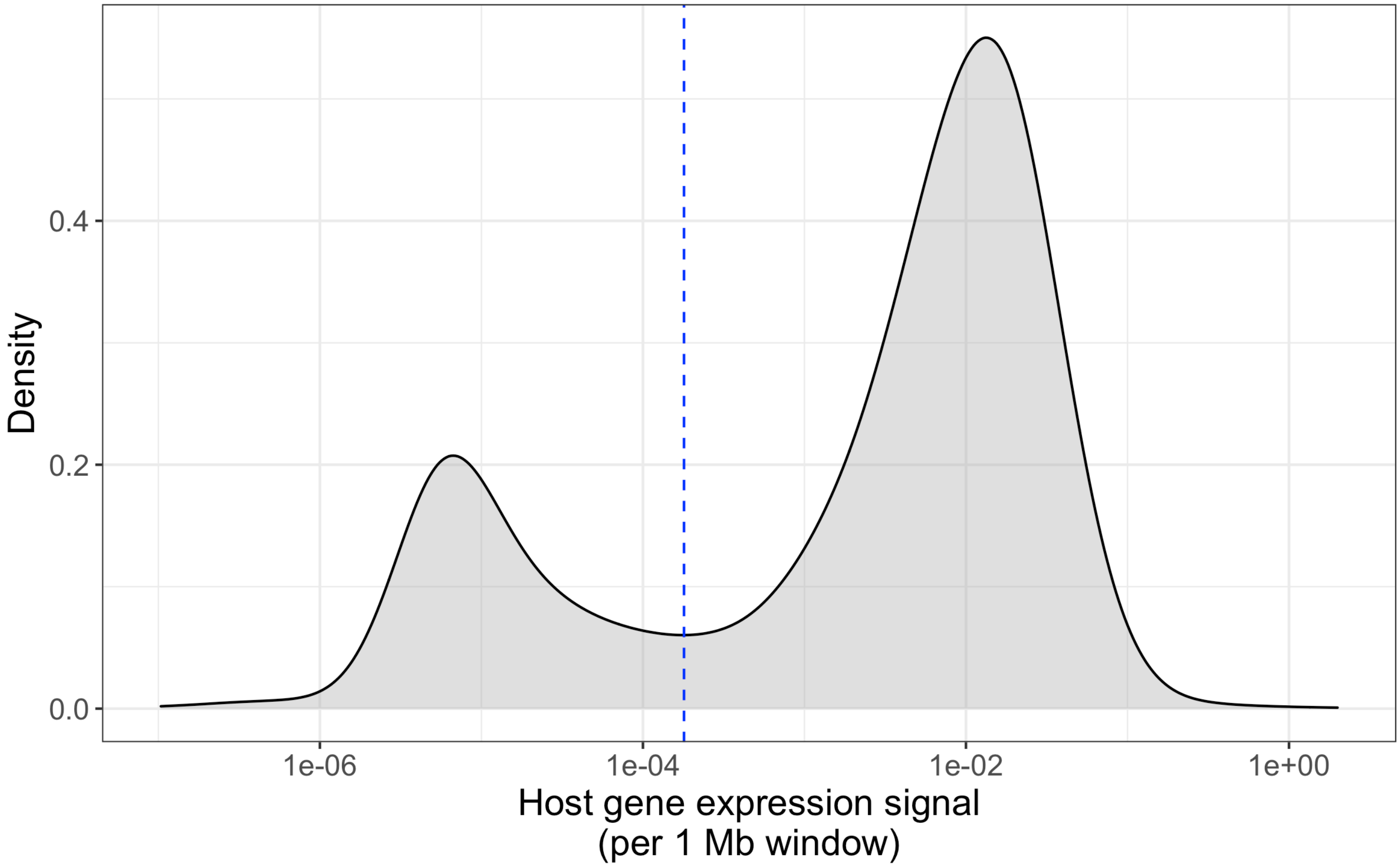

Fig. S13. Counterselection of HTLV-1 proviruses in highly expressing genomic regions*. The average RNA expression intensity per 1 Mb window was quantified across each chromosome. The distribution of RNA expression density (shown on log scale) is bimodal. A local minimum separates genomic regions with high expression from those with low expression.*

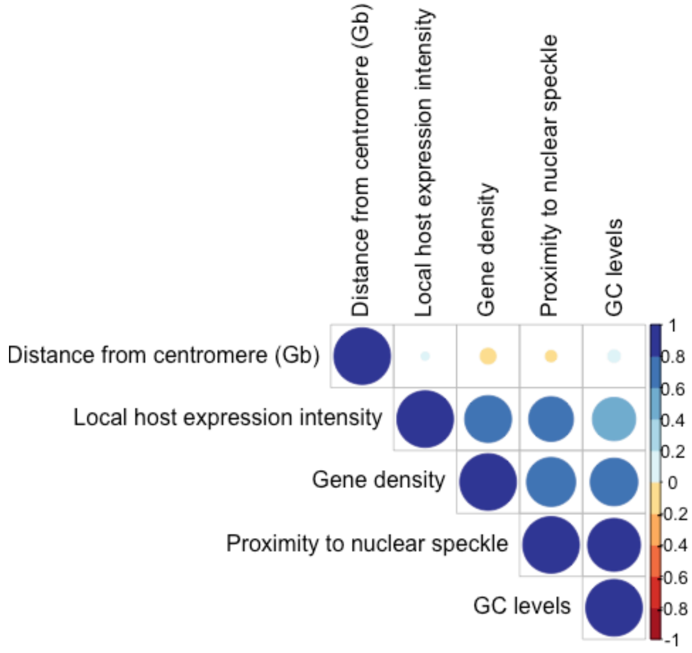

***Fig. S14. Correlation plot, potential predictors of HTLV-1 survival** Each circle represents the magnitude (diameter) and the sign (colour) of the correlation coefficient (Pearson's) between the respective variables.*

	Dataset	Samples	Integration sites	Publication reference
HTLV-1	HTLV1invivo1	142	225425	(9)
	HTLV1invivo2	14	40410	(5)
	HTLV1invivo3	196	6393	(13)
	HTLV1invivo4	36	60350	(36)
	HTLV1invitro1	5	4687	(9)
	HTLV1invitro2	3	4444	(5)
	HTLV1invitro3	8	226019	This manuscript
HIV-1	HIV1invivo1	36	32569	(27)
	HIV1invivo2	25	13142	(27)
	HIV1invitro1	2	65924	(27)
Random	In silico sites	1	109218	This manuscript

Table S1 - Integration site datasets used in this work

Dataset type	file accession	experiment/ project accession	assay name	target	lab	data availability
TFBS	ENCFF003VDB	ENCSR778UBR	ChIP-seq	ARID3A	Michael Snyder, Stanford	www.encodeproject.org
TFBS	ENCFF758RQJ	ENCSR590KEQ	ChIP-seq	ARNT	Michael Snyder, Stanford	www.encodeproject.org
TFBS	ENCFF096XRG	ENCSR849WCQ	ChIP-seq	ASH2L	Bradley Bernstein, Broad	www.encodeproject.org
TFBS	ENCFF806KKM	ENCSR000BQK	ChIP-seq	ATF2	Richard Myers, HAIB	www.encodeproject.org
TFBS	ENCFF495PWL	ENCSR014YCR	ChIP-seq	ATF7	Michael Snyder, Stanford	www.encodeproject.org
TFBS	ENCFF725YZH	ENCSR636MKU	ChIP-seq	BACH1	Michael Snyder, Stanford	www.encodeproject.org
TFBS	ENCFF832YIE	ENCSR000BGT	ChIP-seq	BATF	Richard Myers, HAIB	www.encodeproject.org
TFBS	ENCFF383HAY	ENCSR000BHA	ChIP-seq	BCL11A	Richard Myers, HAIB	www.encodeproject.org
TFBS	ENCFF247MHT	ENCSR000BNQ	ChIP-seq	BCL3	Richard Myers, HAIB	www.encodeproject.org
TFBS	ENCFF587BJK	ENCSR000BJZ	ChIP-seq	BCLAF1	Richard Myers, HAIB	www.encodeproject.org
TFBS	ENCFF370ZNL	ENCSR987MTA	ChIP-seq	BHLHE40	Michael Snyder, Stanford	www.encodeproject.org
TFBS	ENCFF592LPO	ENCSR469WII	ChIP-seq	BMI1	Michael Snyder, Stanford	www.encodeproject.org
TFBS	ENCFF005JKU	ENCSR000DZS	ChIP-seq	BRCA1	Michael Snyder, Stanford	www.encodeproject.org
TFBS	ENCFF070SOX	ENCSR860UHK	ChIP-seq	CBFB	Richard Myers, HAIB	www.encodeproject.org
TFBS	ENCFF552QOA	ENCSR549NPZ	ChIP-seq	CBX3	Bradley Bernstein, Broad	www.encodeproject.org
TFBS	ENCFF417SVR	ENCSR372GIN	ChIP-seq	CBX5	Richard Myers, HAIB	www.encodeproject.org
TFBS	ENCFF786YYI	ENCSR681NOM	ChIP-seq	CEBPB	Michael Snyder, Stanford	www.encodeproject.org
TFBS	ENCFF243GOG	ENCSR347NOB	ChIP-seq	CEBPZ	Michael Snyder, Stanford	www.encodeproject.org
TFBS	ENCFF863CTN	ENCSR000DZE	ChIP-seq	CHD1	Michael Snyder, Stanford	www.encodeproject.org
TFBS	ENCFF546AYN	ENCSR000DZR	ChIP-seq	CHD2	Michael Snyder, Stanford	www.encodeproject.org
TFBS	ENCFF249SIN	ENCSR751CJG	ChIP-seq	CHD4	Bradley Bernstein, Broad	www.encodeproject.org
TFBS	ENCFF091YID	ENCSR839XZU	ChIP-seq	CREM	Richard Myers, HAIB	www.encodeproject.org
TFBS	ENCFF960ZGP	ENCSR000DZN	ChIP-	CTCF	Michael Snyder,	www.encodeproject.org

Dataset type	file accession	experiment/ project accession	assay name	target	lab	data availability
			seq		Stanford	
TFBS	ENCFF567NFS	ENCSR000DYR	ChIP-seq	CUX1	Michael Snyder, Stanford	www.encodeproject.org
TFBS	ENCFF771IAW	ENCSR509FWH	ChIP-seq	DPF2	Michael Snyder, Stanford	www.encodeproject.org
TFBS	ENCFF687SFB	ENCSR000DYY	ChIP-seq	E2F4	Michael Snyder, Stanford	www.encodeproject.org
TFBS	ENCFF412GFI	ENCSR793HVL	ChIP-seq	E2F8	Michael Snyder, Stanford	www.encodeproject.org
TFBS	ENCFF035GFS	ENCSR439WAF	ChIP-seq	E4F1	Michael Snyder, Stanford	www.encodeproject.org
TFBS	ENCFF864NSW	ENCSR000BGU	ChIP-seq	EBF1	Richard Myers, HAIB	www.encodeproject.org
TFBS	ENCFF023ALY	ENCSR199WXF	ChIP-seq	EED	Richard Myers, HAIB	www.encodeproject.org
TFBS	ENCFF948CPI	ENCSR841NDX	ChIP-seq	ELF1	Michael Snyder, Stanford	www.encodeproject.org
TFBS	ENCFF432AQP	ENCSR000DZB	ChIP-seq	ELK1	Michael Snyder, Stanford	www.encodeproject.org
TFBS	ENCFF080HJX	ENCSR000DZG	ChIP-seq	EP300	Michael Snyder, Stanford	www.encodeproject.org
TFBS	ENCFF722LJP	ENCSR000DYQ	ChIP-seq	ESRRA	Michael Snyder, Stanford	www.encodeproject.org
TFBS	ENCFF980VOD	ENCSR000BKA	ChIP-seq	ETS1	Richard Myers, HAIB	www.encodeproject.org
TFBS	ENCFF745ANU	ENCSR626VUC	ChIP-seq	ETV6	Richard Myers, HAIB	www.encodeproject.org
TFBS	ENCFF615NYO	ENCSR000ARD	ChIP-seq	EZH2	Bradley Bernstein, Broad	www.encodeproject.org
TFBS	ENCFF990MTR	ENCSR861JUQ	ChIP-seq	FOXK2	Michael Snyder, Stanford	www.encodeproject.org
TFBS	ENCFF946ACA	ENCSR331HPA	ChIP-seq	GABPA	Richard Myers, HAIB	www.encodeproject.org
TFBS	ENCFF298AIX	ENCSR828NCB	ChIP-seq	GATAD2B	Michael Snyder, Stanford	www.encodeproject.org
TFBS	ENCFF722QBB	ENCSR514VAY	ChIP-seq	HCFC1	Michael Snyder, Stanford	www.encodeproject.org
TFBS	ENCFF299UPZ	ENCSR330OEO	ChIP-seq	HDAC2	Bradley Bernstein, Broad	www.encodeproject.org
TFBS	ENCFF442WRJ	ENCSR145XQO	ChIP-seq	HDGF	Michael Snyder, Stanford	www.encodeproject.org
TFBS	ENCFF603BID	ENCSR009MBP	ChIP-seq	HSF1	Michael Snyder, Stanford	www.encodeproject.org
TFBS	ENCFF018NNF	ENCSR441VHN	ChIP-seq	IKZF1	Michael Snyder, Stanford	www.encodeproject.org

Dataset type	file accession	experiment/ project accession	assay name	target	lab	data availability
TFBS	ENCFF088OLI	ENCSR822AHX	ChIP-seq	IKZF2	Michael Snyder, Stanford	www.encodeproject.org
TFBS	ENCFF604AZX	ENCSR408JQO	ChIP-seq	IRF3	Michael Snyder, Stanford	www.encodeproject.org
TFBS	ENCFF720YMW	ENCSR000BGY	ChIP-seq	IRF4	Richard Myers, HAIB	www.encodeproject.org
TFBS	ENCFF843HDK	ENCSR976TBC	ChIP-seq	IRF5	Michael Snyder, Stanford	www.encodeproject.org
TFBS	ENCFF478XNA	ENCSR897MMC	ChIP-seq	JUNB	Richard Myers, HAIB	www.encodeproject.org
TFBS	ENCFF873DJD	ENCSR000DYS	ChIP-seq	JUND	Michael Snyder, Stanford	www.encodeproject.org
TFBS	ENCFF710ROZ	ENCSR000DNO	ChIP-seq	KAT2A	Kevin Struhl, HMS	www.encodeproject.org
TFBS	ENCFF799KZP	ENCSR391IWM	ChIP-seq	KDM1A	Bradley Bernstein, Broad	www.encodeproject.org
TFBS	ENCFF417WPC	ENCSR974OFJ	ChIP-seq	KLF5	Michael Snyder, Stanford	www.encodeproject.org
TFBS	ENCFF305SLO	ENCSR657PEW	ChIP-seq	LARP7	Michael Snyder, Stanford	www.encodeproject.org
TFBS	ENCFF186AWV	ENCSR000DYV	ChIP-seq	MAFK	Michael Snyder, Stanford	www.encodeproject.org
TFBS	ENCFF270NAL	ENCSR000DZF	ChIP-seq	MAX	Michael Snyder, Stanford	www.encodeproject.org
TFBS	ENCFF348STZ	ENCSR000DZA	ChIP-seq	MAZ	Michael Snyder, Stanford	www.encodeproject.org
TFBS	ENCFF958GXF	ENCSR000BKB	ChIP-seq	MEF2A	Richard Myers, HAIB	www.encodeproject.org
TFBS	ENCFF623FAW	ENCSR177VFS	ChIP-seq	MEF2B	Michael Snyder, Stanford	www.encodeproject.org
TFBS	ENCFF830BRO	ENCSR000BNG	ChIP-seq	MEF2C	Richard Myers, HAIB	www.encodeproject.org
TFBS	ENCFF125MEN	ENCSR552XSN	ChIP-seq	MLLT1	Michael Snyder, Stanford	www.encodeproject.org
TFBS	ENCFF587POH	ENCSR293QAR	ChIP-seq	MTA2	Michael Snyder, Stanford	www.encodeproject.org
TFBS	ENCFF661FMB	ENCSR000BRH	ChIP-seq	MTA3	Richard Myers, HAIB	www.encodeproject.org
TFBS	ENCFF199HGX	ENCSR000DZI	ChIP-seq	MXI1	Michael Snyder, Stanford	www.encodeproject.org
TFBS	ENCFF402TSJ	ENCSR819ATC	ChIP-seq	MYB	Michael Snyder, Stanford	www.encodeproject.org
TFBS	ENCFF811VEN	ENCSR278SQL	ChIP-seq	NBN	Michael Snyder, Stanford	www.encodeproject.org
TFBS	ENCFF138ZBJ	ENCSR000BQL	ChIP-	NFATC1	Richard Myers,	www.encodeproject.org

Dataset type	file accession	experiment/ project accession	assay name	target	lab	data availability
			seq		HAIB	
TFBS	ENCFF704PDA	ENCSR437GBJ	ChIP-seq	NFATC3	Michael Snyder, Stanford	www.encodeproject.org
TFBS	ENCFF480WDX	ENCSR000BRN	ChIP-seq	NFIC	Richard Myers, HAIB	www.encodeproject.org
TFBS	ENCFF860IXB	ENCSR746XEG	ChIP-seq	NFXL1	Michael Snyder, Stanford	www.encodeproject.org
TFBS	ENCFF278GJK	ENCSR000DNN	ChIP-seq	NFYA	Kevin Struhl, HMS	www.encodeproject.org
TFBS	ENCFF510NDO	ENCSR000DNM	ChIP-seq	NFYB	Kevin Struhl, HMS	www.encodeproject.org
TFBS	ENCFF084NXU	ENCSR732PJX	ChIP-seq	NKRF	Michael Snyder, Stanford	www.encodeproject.org
TFBS	ENCFF462AKP	ENCSR784VIQ	ChIP-seq	NR2C1	Michael Snyder, Stanford	www.encodeproject.org
TFBS	ENCFF434HVY	ENCSR000EUL	ChIP-seq	NR2C2	Peggy Farnham, USC	www.encodeproject.org
TFBS	ENCFF531KOV	ENCSR514VYD	ChIP-seq	NR2F1	Michael Snyder, Stanford	www.encodeproject.org
TFBS	ENCFF652BRY	ENCSR000DZO	ChIP-seq	NRF1	Michael Snyder, Stanford	www.encodeproject.org
TFBS	ENCFF946SAG	ENCSR000BHJ	ChIP-seq	PAX5	Richard Myers, HAIB	www.encodeproject.org
TFBS	ENCFF992JWY	ENCSR192AFN	ChIP-seq	PAX8	Michael Snyder, Stanford	www.encodeproject.org
TFBS	ENCFF926LHG	ENCSR000BGR	ChIP-seq	PBX3	Richard Myers, HAIB	www.encodeproject.org
TFBS	ENCFF335ADU	ENCSR711XNY	ChIP-seq	PKNOX1	Michael Snyder, Stanford	www.encodeproject.org
TFBS	ENCFF455ZLJ	ENCSR000BGD	ChIP-seq	POLR2A	Richard Myers, HAIB	www.encodeproject.org
TFBS	ENCFF847DXY	ENCSR000DZK	ChIP-seq	POLR2AphosphoS2	Michael Snyder, Stanford	www.encodeproject.org
TFBS	ENCFF600GQL	ENCSR000BIF	ChIP-seq	POLR2AphosphoS5	Richard Myers, HAIB	www.encodeproject.org
TFBS	ENCFF654EGO	ENCSR000BMY	ChIP-seq	RAD21	Richard Myers, HAIB	www.encodeproject.org
TFBS	ENCFF996NBR	ENCSR482TWQ	ChIP-seq	RAD51	Michael Snyder, Stanford	www.encodeproject.org
TFBS	ENCFF034OSV	ENCSR785OKZ	ChIP-seq	RB1	Michael Snyder, Stanford	www.encodeproject.org
TFBS	ENCFF687SSY	ENCSR330EXS	ChIP-seq	RBBP5	Bradley Bernstein, Broad	www.encodeproject.org
TFBS	ENCFF470ZMK	ENCSR000DZC	ChIP-seq	RCOR1	Michael Snyder, Stanford	www.encodeproject.org

Dataset type	file accession	experiment/ project accession	assay name	target	lab	data availability
TFBS	ENCFF105YDI	ENCSR387QUV	ChIP-seq	RELB	Michael Snyder, Stanford	www.encodeproject.org
TFBS	ENCFF313CII	ENCSR000BQS	ChIP-seq	REST	Richard Myers, HAIB	www.encodeproject.org
TFBS	ENCFF259LNG	ENCSR000DZW	ChIP-seq	RFX5	Michael Snyder, Stanford	www.encodeproject.org
TFBS	ENCFF677QUK	ENCSR000BRI	ChIP-seq	RUNX3	Richard Myers, HAIB	www.encodeproject.org
TFBS	ENCFF313BDA	ENCSR000BJD	ChIP-seq	RXRA	Richard Myers, HAIB	www.encodeproject.org
TFBS	ENCFF050CYK	ENCSR000DYX	ChIP-seq	SIN3A	Michael Snyder, Stanford	www.encodeproject.org
TFBS	ENCFF864TFH	ENCSR000BJE	ChIP-seq	SIX5	Richard Myers, HAIB	www.encodeproject.org
TFBS	ENCFF903KEI	ENCSR212YKD	ChIP-seq	SKIL	Michael Snyder, Stanford	www.encodeproject.org
TFBS	ENCFF987PGY	ENCSR813DCK	ChIP-seq	SMAD1	Michael Snyder, Stanford	www.encodeproject.org
TFBS	ENCFF855SJG	ENCSR251OVJ	ChIP-seq	SMAD5	Richard Myers, HAIB	www.encodeproject.org
TFBS	ENCFF052STI	ENCSR706YUH	ChIP-seq	SMARCA5	Michael Snyder, Stanford	www.encodeproject.org
TFBS	ENCFF572RPI	ENCSR000DZP	ChIP-seq	SMC3	Michael Snyder, Stanford	www.encodeproject.org
TFBS	ENCFF071ZMW	ENCSR000BGQ	ChIP-seq	SPI1	Richard Myers, HAIB	www.encodeproject.org
TFBS	ENCFF766WWB	ENCSR041XML	ChIP-seq	SRF	Michael Snyder, Stanford	www.encodeproject.org
TFBS	ENCFF323QQU	ENCSR332EYT	ChIP-seq	STAT1	Michael Snyder, Stanford	www.encodeproject.org
TFBS	ENCFF923CHO	ENCSR000DZV	ChIP-seq	STAT3	Michael Snyder, Stanford	www.encodeproject.org
TFBS	ENCFF383YEA	ENCSR000BQZ	ChIP-seq	STAT5A	Richard Myers, HAIB	www.encodeproject.org
TFBS	ENCFF069YVD	ENCSR000DNP	ChIP-seq	SUPT20H	Kevin Struhl, HMS	www.encodeproject.org
TFBS	ENCFF540AAP	ENCSR000BGS	ChIP-seq	TAF1	Richard Myers, HAIB	www.encodeproject.org
TFBS	ENCFF668JHK	ENCSR412QBS	ChIP-seq	TARDBP	Michael Snyder, Stanford	www.encodeproject.org
TFBS	ENCFF392JWA	ENCSR000DYZ	ChIP-seq	TBL1XR1	Michael Snyder, Stanford	www.encodeproject.org
TFBS	ENCFF896UZB	ENCSR000DZZ	ChIP-seq	TBP	Michael Snyder, Stanford	www.encodeproject.org
TFBS	ENCFF971VHK	ENCSR739IHN	ChIP-	TBX21	Michael Snyder,	www.encodeproject.org

Dataset type	file accession	experiment/ project accession	assay name	target	lab	data availability
			seq		Stanford	
TFBS	ENCFF768VSH	ENCSR000BGZ	ChIP-seq	TCF12	Richard Myers, HAIB	www.encodeproject.org
TFBS	ENCFF152RNE	ENCSR501DKS	ChIP-seq	TCF7	Richard Myers, HAIB	www.encodeproject.org
TFBS	ENCFF552WAH	ENCSR835XKS	ChIP-seq	TRIM22	Michael Snyder, Stanford	www.encodeproject.org
TFBS	ENCFF295ZLM	ENCSR459FTB	ChIP-seq	UBTF	Michael Snyder, Stanford	www.encodeproject.org
TFBS	ENCFF514SWA	ENCSR000DZU	ChIP-seq	USF2	Michael Snyder, Stanford	www.encodeproject.org
TFBS	ENCFF514DDI	ENCSR000EAA	ChIP-seq	WRNIP1	Michael Snyder, Stanford	www.encodeproject.org
TFBS	ENCFF500RBO	ENCSR205SKQ	ChIP-seq	YBX1	Michael Snyder, Stanford	www.encodeproject.org
TFBS	ENCFF223MUF	ENCSR000BNP	ChIP-seq	YY1	Richard Myers, HAIB	www.encodeproject.org
TFBS	ENCFF630FLK	ENCSR207PFI	ChIP-seq	ZBED1	Richard Myers, HAIB	www.encodeproject.org
TFBS	ENCFF475DID	ENCSR542FLV	ChIP-seq	ZBTB33	Michael Snyder, Stanford	www.encodeproject.org
TFBS	ENCFF084IUW	ENCSR189YYK	ChIP-seq	ZBTB40	Michael Snyder, Stanford	www.encodeproject.org
TFBS	ENCFF224WII	ENCSR900XDB	ChIP-seq	ZFP36	Michael Snyder, Stanford	www.encodeproject.org
TFBS	ENCFF193POQ	ENCSR000DZL	ChIP-seq	ZNF143	Michael Snyder, Stanford	www.encodeproject.org
TFBS	ENCFF676BIG	ENCSR117KWH	ChIP-seq	ZNF207	Michael Snyder, Stanford	www.encodeproject.org
TFBS	ENCFF200SLC	ENCSR764CZW	ChIP-seq	ZNF217	Michael Snyder, Stanford	www.encodeproject.org
TFBS	ENCFF313HBL	ENCSR072PWP	ChIP-seq	ZNF24	Michael Snyder, Stanford	www.encodeproject.org
TFBS	ENCFF942MDT	ENCSR000DYP	ChIP-seq	ZNF384	Michael Snyder, Stanford	www.encodeproject.org
TFBS	ENCFF615DTQ	ENCSR173ZVL	ChIP-seq	ZNF592	Michael Snyder, Stanford	www.encodeproject.org
TFBS	ENCFF777DVJ	ENCSR075FNZ	ChIP-seq	ZNF622	Michael Snyder, Stanford	www.encodeproject.org
TFBS	ENCFF137BRA	ENCSR859FDL	ChIP-seq	ZNF687	Michael Snyder, Stanford	www.encodeproject.org
TFBS	ENCFF214NJL	ENCSR412YGM	ChIP-seq	ZSCAN29	Michael Snyder, Stanford	www.encodeproject.org
TFBS	ENCFF260NAX	ENCSR000DNQ	ChIP-seq	ZZZ3	Kevin Struhl, HMS	www.encodeproject.org

Dataset type	file accession	experiment/ project accession	assay name	target	lab	data availability
HistMarks	ENCFF831ZHL	ENCSR000AKF	ChIP-seq	H3K4me1	Bradley Bernstein, Broad	www.encodeproject.org
HistMarks	ENCFF039HDL	ENCSR000AKG	ChIP-seq	H3K4me2	Bradley Bernstein, Broad	www.encodeproject.org
HistMarks	ENCFF039JOT	ENCSR000DRX	ChIP-seq	H3K27me3	John Stamatoyannopoulos, UW	www.encodeproject.org
HistMarks	ENCFF340JIF	ENCSR000AKC	ChIP-seq	H3K27ac	Bradley Bernstein, Broad	www.encodeproject.org
HistMarks	ENCFF028KBY	ENCSR000AKH	ChIP-seq	H3K9ac	Bradley Bernstein, Broad	www.encodeproject.org
HistMarks	ENCFF392LMQ	ENCSR000AOX	ChIP-seq	H3K9me3	Bradley Bernstein, Broad	www.encodeproject.org
HistMarks	ENCFF171MDW	ENCSR000DRW	ChIP-seq	H3K36me3	John Stamatoyannopoulos, UW	www.encodeproject.org
HistMarks	ENCFF601YET	ENCSR000AOV	ChIP-seq	H2AFZ	Bradley Bernstein, Broad	www.encodeproject.org
HistMarks	ENCFF003DXG	ENCSR057BWO	ChIP-seq	H3K4me3	Bradley Bernstein, Broad	www.encodeproject.org
HistMarks	ENCFF309OEW	ENCSR000AKI	ChIP-seq	H4K20me1	Bradley Bernstein, Broad	www.encodeproject.org
HistMarks	ENCFF803DJF	ENCSR000AOW	ChIP-seq	H3K79me2	Bradley Bernstein, Broad	www.encodeproject.org
HistMarks	ENCFF327BUB	ENCSR702RZM	ChIP-seq	H3K27me3	Bradley Bernstein, Broad	www.encodeproject.org
HistMarks	ENCFF094AZY	ENCSR117IQD	ChIP-seq	H3K9me3	Bradley Bernstein, Broad	www.encodeproject.org
HistMarks	ENCFF986YKG	ENCSR887ESB	ChIP-seq	H3K4me1	Bradley Bernstein, Broad	www.encodeproject.org
HistMarks	ENCFF652DFI	ENCSR580WRV	ChIP-seq	H3K36me3	Bradley Bernstein, Broad	www.encodeproject.org
DHS	ENCFF598KWZ	ENCSR000EMT	DNase-seq	NA	John Stamatoyannopoulos, UW	www.encodeproject.org
DHS	ENCFF073ORT	ENCSR000EMT	DNase-seq	NA	John Stamatoyannopoulos, UW	www.encodeproject.org
DHS	ENCFF749FDY	ENCSR239XNU	DNase-seq	NA	John Stamatoyannopoulos, UW	www.encodeproject.org
TSAseq	SRR5516303	PRJNA321975	TSA-seq	SON - sample	Belmont, UIUC	www.ebi.ac.uk/ena/
TSAseq	SRR5516304	PRJNA321975	TSA-seq	SON - control	Belmont, UIUC	www.ebi.ac.uk/ena/
TSAseq	SRR7065754	PRJNA321975	TSA-	Lamin A/C -	Belmont, UIUC	www.ebi.ac.uk/ena/

Dataset type	file accession	experiment/ project accession	assay name	target	lab	data availability
			seq	sample		
TSAseq	SRR7065755	PRJNA321975	TSA-seq	Lamin A/C - control	Belmont, UIUC	www.ebi.ac.uk/ena/
TSAseq	SRR7065760	PRJNA321975	TSA-seq	Lamin B - sample	Belmont, UIUC	www.ebi.ac.uk/ena/
TSAseq	SRR7065761	PRJNA321975	TSA-seq	Lamin B - control	Belmont, UIUC	www.ebi.ac.uk/ena/
LADs	GSE94971	PRJNA374977	DamID-seq	Lamin B1	Schirmer, U. of Edinburgh	www.ncbi.nlm.nih.gov/geo
	ENCFF356LFX	ENCSR636HFF		exclusion list regions	Anshul Kundaje, Stanford	www.encodeproject.org

Table S2 - Genomic annotation datasets used in this work

Predictor	Parameter Estimates	Std. Error	t value	$-log_{10}(p.value)$	R^2	adjusted R^2
Local host expression intensity	-0.08	0.00	-28.44	154.97	0.23	0.23
Proximity to nuclear speckle	-0.38	0.01	-28.17	152.36	0.23	0.23
GC levels	-2.14	0.08	-26.99	141.34	0.21	0.21
Gene density	-0.27	0.01	-23.15	107.47	0.17	0.17
Distance from centromere (Gb)	-4.92	0.28	-17.75	66.02	0.11	0.11

Table S3 - Univariate linear regression, potential predictors of HTLV-1 survival

Predictor	Parameter Estimates	Std. Error	t value	$-log_{10}(p.value)$	R^2	adjusted R^2
Proximity to nuclear speckle	-0.32	0.03	-11.89	30.87	0.06	0.06
GC levels	-1.83	0.16	-11.65	29.73	0.06	0.06
Gene density	-0.23	0.02	-9.48	20.18	0.04	0.04
Local host expression intensity	-0.06	0.01	-8.21	15.41	0.03	0.03
Distance from centromere (Gb)	-1.44	0.52	-2.79	2.27	0.00	0.00

Table S4 - Univariate linear regression, potential predictors of HIV-1 survival

Supplementary Data S1 - Details of integration sites used in this work

For each site, the chromosome, chromosomal position (hg38 human genome reference) and orientation are listed.
